# Physiological impact of portable air filtration systems on male‐pediatric cardiovascular health amid hazardous air pollution

**DOI:** 10.14814/phy2.70517

**Published:** 2025-08-20

**Authors:** Geetika Arya, Rahul Rulia, Ashwani Kumar, Aman Ahuja, Rashmi Bhardwaj, Vineela Surapaneni, Vishal Raj, Jyothi Geetha Mohankumar, Samruddhi Chougale, Dhruva Chaudhry, Pawan Kumar Singh

**Affiliations:** ^1^ Pandit Bhagwat Dayal Sharma Post Graduate Institute of Medical Sciences Rohtak India; ^2^ Maharishi Dayanand University Rohtak India

**Keywords:** air pollution, air purifiers, cardiovascular, pediatric subjects, PM2.5

## Abstract

Air pollution is a public health crisis worldwide, significantly affecting low‐ and middle‐income countries. This study evaluated the physiological impact of hazardous‐category air pollution and the role of portable air filtration systems (AFS) on cardiovascular and inflammatory health of pediatric subjects during peakpollution season. In this non‐randomized, controlled study conducted at a boys' orphanage in North India, 32 boys were subjected to the use of AFS (only during sleep time) for a 2‐week period. Measurements included blood pressure, pulse rate, C‐Reactive Protein, 8‐oxo‐deoxyguanosine levels, and pulse wave velocity (PWV) at three points: baseline, 2 weeks post‐AFS installation, and 2 weeks after sham filtration (high‐efficiency‐filters removed). Mean age was 12.94 ± 2.6 years. Baseline PM2.5 levels were 484 ± 26.8 μg/m^3^, which improved significantly to 125.25 ± 16.6 μg/m^3^ with AFS use (*p* < 0.001). Blood pressure (both systolic as well as diastolic) and pulse wave velocity showed statistically significant improvements after AFS use, with inflammatory and DNA damage biomarkers also reducing markedly (*p* < 0.05). After shifting from true AFS to sham AFS, the blood pressures, pulse wave velocity, CRP levels, and 8‐oxo‐deoxyguanosine levels reached back to the baseline (*p* < 0.05). The study highlights the potential of portable AFS to improve air quality and reduce cardiovascular impact on boys exposed to hazardous air pollution.

## 1 INTRODUCTION

Air pollution is a public health emergency around the globe. Low‐ and middle‐income countries are among those maximally impacted by deteriorating quality of air. Contamination of air by particulate matter and gases like sulfur dioxide, nitrogen dioxide, and others leads to several acute as well as long‐term impacts on health. The particulate matter component of air pollution has been evaluated in several studies in the past for its direct and indirect effects on health. Air pollution attributable cancer and stroke are among the top three factors contributing to age‐standardized mortality rates (Sang et al., 2022). Studies evaluating the impact of particulate matter (PM) on the incidence of cancers and cardiovascular mortality have focused on several modeling strategies to investigate the true impact. Despite the predictive methodologies and the use of retrospective satellite‐based prototypes, all studies have consistently demonstrated the rising contribution of PM toward disease burden (Chen et al., 2024; Sun et al., 2023). The maximally studied health aspect in relation to air pollution has been the risk of cancer and cardiovascular health. In a study by Liu et al., it was demonstrated in an in vitro experiment that exposure to PM2.5 on bronchial epithelial cell lines was associated with reduced viability, increased apoptosis, and a rise in DNA damage markers. The authors also found that *Rad51* gene expression was downregulated in PM2.5‐treated cells. The *Rad51* gene is a key component of DNA repair machinery (Liu et al., 2020). Similarly, in a real‐world cross‐sectional study on 768 subjects, the markers of DNA damage and lipid peroxidation in the urine were found to be significantly correlated with PM2.5 and polycyclic aromatic hydrocarbon levels (Hu et al., 2021).

Cardiovascular impact of air pollution has also been extensively studied and shown to have a linear, positive, and strong correlation. The incidence of stroke, coronary artery diseases, and mortality are known to be associated with particulate matter levels (de Bont et al., 2022). In spite of the extensive literature evidence studying the impact of particulate matter air pollution on cardiovascular and genetic health, there are limited articles focusing on subjects in the pediatric age group. Most articles that have reported the impact of air pollution on the pediatric age group have focused on the respiratory complications only. In a study by Li et al., hospital records of emergency visits by pediatric subjects for respiratory ailments were correlated with levels of ambient ultrafine particulate levels. Relative risk for asthma, pneumonia, upper respiratory tract infection, and bronchitis‐related emergency department visits all were found to be significantly associated with levels of ultrafine particles (Risk Ratio varying from 1.09 to 1.35) (Li et al., 2021). In another study, mild asthma subjects were compared with severe asthma cases, and the exacerbation rates during seasons with spikes in air pollution were found to be significantly higher among the severe cases, with an odds ratio of 1.37 (Kelchtermans et al., 2024). Similar results were also presented in other articles where the association of particulate matter air pollution with increased respiratory emergency‐related visits to hospitals among the pediatric population was highlighted (Glick et al., 2019; Liu et al., 2022). However, the research on the impact of particulate matter air pollution on pediatric cardiovascular and DNA health has been sparse. Additionally, the impact of hazardous category air pollution on health has never been studied. In northern India, the PM2.5 levels are consistently in the ranges of unhealthy to hazardous categories throughout the year. The magnitude of the physiological impact of such poor air quality on the pediatric population's cardiovascular health can be multifold due to several reasons. Firstly, children are frequently exposed to outdoor environments such as at schools, playgrounds, and other activities. Secondly, their higher respiratory and pulse rates, along with greater minute ventilation relative to body weight, make them more susceptible to inhaling and circulating larger amounts of particulate matter compared to adults. Lastly, prolonged exposure to poor air quality may interfere with their normal physiological development.

In order to counter the impact of air pollution, air filtration systems, especially the portable ones (also known as air purifiers) are commercially promoted to be the go‐to devices. Air filtration systems (AFS) work on the principles of adsorptions and mechanical filtration. Activated carbon absorbs the gaseous and ultrafine particulate matter whereas high efficiency particulate air (HEPA) filters entrap particles above the size of 1 micron. Several studies done on air filtration systems have demonstrated their beneficial impact on cardiovascular health (Klaver et al., 2023; Morishita et al., 2018). However, there are contradictory studies which have questioned the real‐world utility of air purifiers, especially in averting the pulmonary impact of air pollution (Shao et al., 2017; Yoda et al., 2020). One concern regarding the effectiveness of air filtration systems is the uncertainty around the optimal duration of exposure needed to benefit from them. Most commonly the air filtration systems are used during nighttime or while being indoors. Children on the other hand, spend much of their day time in outdoor activities that make them susceptible to risk of exposure to particulate matter. Therefore, it remains unknown if the limited night‐time only use of air purifiers can have a beneficial impact on cardiovascular physiology.

To evaluate the impact of air pollution on cardiovascular outcomes in children and to investigate the potential effectiveness of air filtration systems in mitigating these effects, we conducted a prospective study in northern India.

## 2 MATERIALS AND METHODS

### 2.1 Study setting and design

It was a prospective study conducted at a single orphanage for boys. The orphanage is located in a north Indian city which has been on the list of the most polluted cities in the world (Rohtak, Haryana). Subject population consisted of boys residing in the home below the age of 18 years. Most assessments were made in the orphanage whereas blood and other investigations were conducted at a nearby public sector university teaching hospital. The study was cleared by the institutional review board and “Biomedical Research and Ethics Committee” of the institute vide letter number UHSR/RPSAC/2023/148‐149 dated 13‐10‐2023. The study was conducted during the Winter season starting before Diwali and extending to few weeks after New Year (from November 2023 to January 2024).

### 2.2 Ethical considerations

We intended to select only pediatric subjects due to the relative lack of knowledge on the subject of air pollution and cardiovascular risk among this population. It was anticipated that the information gained by this research on pediatric subjects could help in influencing the policymakers towards more stringent steps to curb the risk of air pollution to the children. The study was planned to be conducted on pediatric subjects who lived in a setting where a group of children could remain exposed to the same quality of air (with or without air filtration systems). Such a setting could have been possible only in an orphanage or dormitories or hostels. At first, the feasibility of hostels was sought; however, majority hostels were rejected due to individual rooms with no more than two inhabitants. Dormitories included only subjects above the age of 18 years. Finally, an orphanage was selected as the only feasible site for the study. The study was designed accordingly after detailed consultations with the authorities of the orphanage, which included the legally acceptable representatives. Upon the approval of the authorities, the final protocol was submitted to the institutional review board and later to the biomedical research and ethics committee. The governing council of the nongovernment organization (running the orphanage) was the legal guardian of the residents as per the Indian laws and regulations. For the informed consent requirements, the caretaker of the establishment was appointed by the member‐secretary of the council to sign as LAR (legally acceptable representative). The proposed informed consent process was presented at the BREC meeting, which included taking assent from the participants along with the voluntary consent by the LAR. The assent document was in the local language with images of AFS, blood pressure, and blood sampling procedure and, lastly, the ultrasound procedure. For boys with mild intellectual disability, two additional steps were recommended by the ethics committee—addition of an impartial witness and audiovisual (AV) recording. AV consenting for all boys was waived given the type of intervention involved and nature of the study. All interventions (installation of AFS, blood sampling, and vital examination) were done in the presence of the orphanage staff and principal investigators. Some of the investigations (pulse wave velocity) required boys to be taken to hospital, for which we randomly selected from only those who provided explicit assent and separate written informed consent from the LAR. No child with intellectual limitations was considered for echocardiography. The caretaker and one other staff member from the orphanage accompanied the boys to the hospital (in two omnivans). All informed consents and assents processes were in documents in the ICF narrations. The study was conducted in compliance with ICMR National Ethical Guidelines and the Declaration of Helsinki.

### 2.3 Study participants

The orphanage where the study was conducted is a philanthropic effort of a nongovernment organization for boys who were either orphans, abandoned, or suffering from mild intellectual disability. Only boys below the age of 18 years were accepted in the facility. The building of the orphanage consists of hall‐type dorm rooms, which accommodated 8–10 beds with adjoining cabinets. There were smaller rooms on other floors as well. Additionally, there were areas for the playground, school, and mess. Each hall was approximately 60 square meters in size, with two doors (on same wall) and two windows (on opposite wall of doors). Average night sleeping time included 8–10 h. The remaining hours of the day were spent in schooling, playground, and activities related to the kitchen and housekeeping. Four main halls with maximum capacity were selected for the study. Boys who were assigned to the four main halls were approached for study inclusion. The manager and caretakers were asked specifically not to interchange the allocated rooms during the study period. Informed consent to conduct the study was obtained from the manager, and assent form affirmation was obtained from individual study subjects.

### 2.4 Study procedures

Following consent, 34 boys were selected, residing in four main halls. At the baseline visit, anthropometric parameters, vitals, and other baseline assessments including serological assessments (samples for CRP and 8‐oxo DG) were done. Twelve randomly selected boys from different rooms underwent 2D echocardiography for pulse wave velocity measurement in the study hospital. Following the baseline visit, two portable air filtration systems (AFS) were installed in each room. Air filtration systems were installed in the corner away from the door, along with air quality monitors. Caretakers were encouraged to keep the door and windows closed during sleeping hours, and all subjects were instructed accordingly. Filtration systems and monitors were kept in a switched ON state continuously, and both participants and caretakers were instructed not to change the settings. Daily random inspection visits by the study team were made to the orphanage to ensure compliance and restress the need to keep the doors and windows closed for optimum functioning of the devices. Fifteen (with +5 days window) after the installation, the second visit was conducted to collect the same data as in visit 1. The same subjects (who were selected at the baseline) were again taken to the hospital for echocardiography. Once all second visit activities were completed, filters from AFS were removed (sham‐filtration phase). After 15 days (with +5 days window) of withdrawing AFS, the same assessments were made at the third visit, and echocardiography was done for the same subjects. Real‐time data from air quality monitors was collected and averaged for the preceding 5 days and was used for analysis.

### 2.5 Study intervention

#### 2.5.1 Air filtration systems

We used Eureka Forbes Air Purifier 355® systems which work with HEPA filters and are ideal for 480 square feet rooms. It has four filters‐ activated carbon, plasma, HEPA, and sieve filtration. The H13 HEPA filter is able to clear 99.97% of PM0.1 particles. It was a noiseless device and was kept on auto mode to change fan speed based on real‐time PM2.5 levels. Sham filtration was used in the second phase of the study by removing the HEPA filters from the devices.

#### 2.5.2 Pulse wave velocity

Method of measurement of pulse wave velocity using 2D echocardiography has been described previously (Styczynski et al., 2016). In brief, the distance between the QRS complex on gated ECG waveform and the onset of Doppler waveform was measured at distal aortic arch level and at the level of the left external iliac artery. The distance between the two points was measured over the body surface using a flexible measuring tape (distance between sternal notch, to umbilicus to left groin minus distance between sternal notch and distal aortic arch point). Pulse wave velocity was measured by dividing the distance by the difference of the transit times.

#### 2.5.3 Exposure measurement

Ambient air quality was monitored using SMILEDRIVE® portable devices which are battery operated and use inhale‐fans and laser sensors for reporting particulate matter levels. The device measured several other air quality parameters, but we only recorded PM2.5 and PM10 levels. For analysis, an average of the previous five‐day readings was used.

#### 2.5.4 Serum assessments

Serum samples were separated using a refrigerated centrifuge on the same day of collection and stored at −80°C. Quantitative CRP was measured using the ELISA method (Kit from Q‐line Biotech Ltd. Batch number‐ QLCR230601). Human 8‐oxo‐2′‐deoxyguanosine was measured in serum samples in one go using the ELISA method on kits (ELK8533) by ELK® Biotechnology using manufacturer recommendations (Batch number—ELK10848‐201230‐A1). In brief, the assay uses a competitive inhibition enzyme immunoassay technique and uses a biotin‐conjugated human‐8‐oxo‐DG specific antibody. The concentration of 8‐oxo‐DG was obtained by comparing the OD of samples to the standard curve.

### 2.6 Data analysis

No formal sample size calculation was attempted as the study population was restricted to one single facility. Data was collected in paper format and entered in an excel sheet on the same day where the data was curated and coded. Clinical, anthropometric, and demographic data was recorded and analyzed. Continuous data was reported using means (with standard deviation) and medians (interquartile rage) whereas categorical data was reported as numbers and percentages. For the comparison of the change in blood pressure parameters, pulse wave velocity, and serum biomarkers at different time points, we used a paired *t*‐test, whereas for establishing the correlation between these measurements and PM2.5 levels at different time points, we used Pearson correlation analysis. Statistical analysis was performed on the statistical package for social sciences® version 26 by IBM. CONSORT guidelines by EQUATOR Network were adhered to during the writing of this manuscript (Schulz et al., 2010).

## 3 RESULTS

The study was conducted in the winters from November 2023 to January 2024 at an orphanage located on a state highway in northern India. At the time of the initial visit, 34 boys resided in four halls, which were selected for the installation of air purifiers. Over the course of the study, two were shifted out of the orphanage for nonstudy‐related reasons. Among the remaining 32 boys, the mean age was 12.94 ± 2.6 years. Four boys were known to be suffering from mild intellectual disability but were otherwise free from any physical illness. None of the boys, at the beginning of the study, reported any symptoms or showed any abnormal physical sign (except pallor in 5) suggestive of any ongoing illness. After a thorough physical examination of all subjects, baseline study‐related assessments were made. The mean body mass index at the baseline was 19.17 ± 5.9 kg/m^2^. The mean systolic blood pressure and mean diastolic blood pressure at the time of visit 1 were 109.19 ± 13.9 mmHg and 68.88 ± 11.1 mmHg, respectively. Throughout the study, the mean room air oxygen saturation was above 98%. At baseline visit, PM2.5 and PM10 levels were 484 ± 26.8 and 251.75 ± 30.9 μg/m^3^, respectively (Hazardous category as per Unites States Environmental Protection Agency). Despite continuous use of the air filtration system, the lowest indoor levels of PM2.5 did not reach WHO recommended limits. The best recorded levels were 125.25 ± 16.6 μg/m^3^ at visit 2. Detailed baseline data is presented in Table 1.

**TABLE 1:** Baseline demographic variables of the study population at visit 1.

Variables		Study population (mean ± SD)
Age (years)		12.94 ± 2.6
Weight (kilograms)		33.97 ± 8.3
BMI (kilogram/meter^2^)		19.17 ± 5.9
Baseline blood pressure	Mean SBP (mmHg)	109.19 ± 13.9
	Mean DBP (mmHg)	67.88 ± 11.1
Pulse rate (per minute)		97.31 ± 16.0
Oxygen saturation (%)		98.75 ± 1.1
Baselines air pollution parameters	PM2.5 (μg/m^3^)	484 ± 26.8
	PM10 (μg/m^3^)	251.75 ± 30.9

Abbreviations: BMI, body mass index; DBP, diastolic blood pressur; MAP, mean arterial pressure; PM10, particulate matter of diameter 10 μm or less; PM2.5, particulate matter of diameter 2.5 μm or les; SBP, systolic blood pressure; SD, standard deviation.

Using paired *t*‐test analysis, it was found that both fall and rise in PM2.5 and PM10 levels from visit 1 to 2 and from visit 2 to 3 (respectively) were statistically significant. Mean systolic blood pressure at visit 2 was 102.06 ± 10.6 mmHg, resulting in a mean 7.13 mmHg fall from visit 1, which was statistically significant (*p* < 0.001). Similarly, the rise in SBP at visit 3 was statistically significant (*p* = 0.003). A similar statistically significant trend was also followed for diastolic blood pressure. Pulse rate also showed a significant fall from visit 1 to 2 (97.31 ± 16 vs. 89.81 ± 14.3 per minute, *p* < 0.001). At visit 3, pulse rate rose back to the visit 1 baseline value (97.88 ± 13.3) per minute. Oxygen saturation values, as measured by pulse oximetry, did not show any significant change between the three visits. CRP and 8‐oxo‐2′deoxyguanosine levels data were not available for a few subjects due to sample processing errors. However, the available data also followed a similar trend to that of blood pressure. Both rise and fall for both serological parameters between the three visits were statistically significant.

**FIGURE 1:**
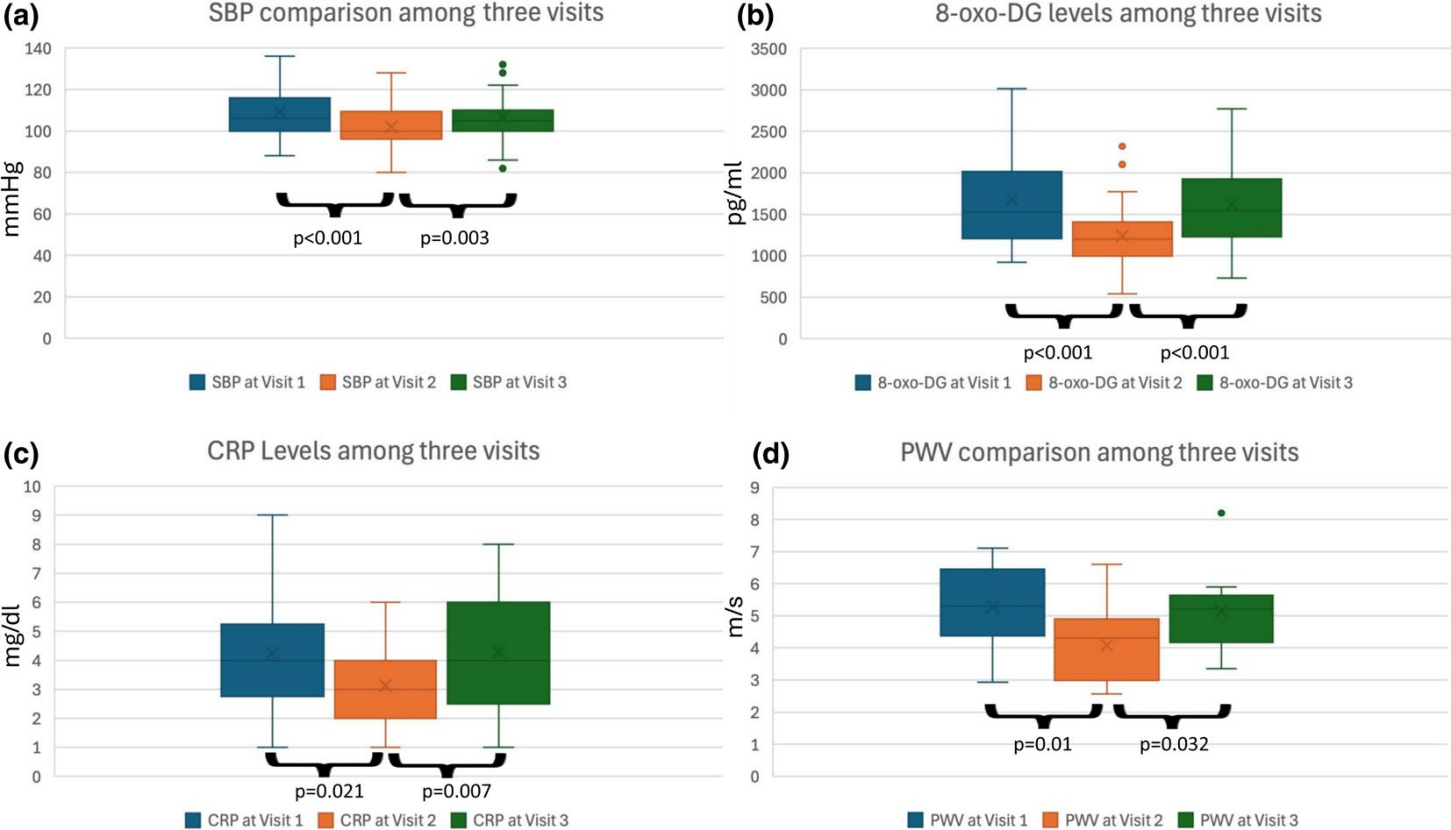
Plots showing significant differences in (a) systolic blood pressure (SBP), (b) 8‐oxo‐DG, (c) CRP levels between three visits. (d) Line diagram showing PWV trend (mean values) of each subject (color coded) between three visits. (a)–(c) show box and whisker plots with boxes representing the interquartile range and whiskers showing the overall range of the values. 8‐oxo‐DG, 8‐Oxo‐2′‐deoxyguanosine; CRP, C reactive protein; PWV, pulse wave velocity.

Pulse wave velocity was measured at each visit on 11 boys. All 11 boys were transported to the hospital for recording of pulse wave velocity. The baseline pulse wave velocity on visit 1 was 5.28 ± 1.3 m/s. Both fall in pulse wave velocity (from visit 1 to 2) as well as rise (from visit 2 to 3) were statistically significant (*p* = 0.005 and *p* = 0.032, respectively). Detailed data of cardiovascular, inflammatory, and biomarkers of DNA damage among the three visits is presented in Table 2 and Figure 1.

**TABLE 2:** Comparison of outcomes between the three visits.

Variables	Visit 1 (baseline)	Visit 2 (after use of AFS)	Visit 3 (after removal of AFS)	*p* Value (visit 1 vs. visit 2)	*p* Value (visit 2 vs. visit 3)
Systolic Blood Pressure	109.19 ± 13.9	102.06 ± 10.6	106.63 ± 12.0	<0.001	0.003
Diastolic Blood Pressure	67.88 ± 11.1	63.81 ± 8.3	66.44 ± 8.2	0.001	0.004
Pulse Rate	97.31 ± 16.0	89.81 ± 14.3	97.88 ± 13.3	<0.001	<0.001
CRP	4.31 ± 1.9	3.04 ± 1.2	4.21 ± 1.8	0.021	0.007
8‐oxo‐DG	1641.45 ± 571.0	1240.58 ± 398.7	1583.07 ± 476.5	<0.001	<0.001
Pulse wave velocity	5.28 ± 1.3	4.08 ± 1.2	5.14 ± 1.3	0.01	0.032
PM2.5	484 ± 26.8	125.25 ± 16.6	402.5 ± 12.0	<0.001	<0.001
PM10	251.75 ± 30.9	61.75 ± 4.7	248.50 ± 18.5	<0.001	<0.001

*Note*: All values are mean ± SD.

Abbreviations: 8‐oxo‐DG, 8‐Oxo‐2′‐deoxyguanosine; AFS, air filtration system; CRP, C reactive protein; PM10, particulate matter of diameter 10 μm or less; PM2.5: Particulate matter of diameter 2.5 μm or less.

Using the Pearson correlation test, it was found that there was a statistically significant positive correlation between the change in 8‐oxo‐deoxyguanosine levels and the fall in PM2.5 levels between visit 1 and 2 (*r* = 0.428; *p* = 0.041). Similarly, the change in PM10 also had a significant positive correlation with the change in 8‐oxo‐deoxyguanosine levels (*r* = 0.515; *p* = 0.012). The change in pulse wave velocity and the change PM2.5 levels between visit 1 and 2 also had a significant positive correlation among each other (*r* = 0.674; *p* < 0.023). The change in none of the blood pressure parameters (either between visit 1 and 2 or between visit 2 and 3) had any significant correlation with the respective change in PM2.5 or PM10 levels.

## 4 DISCUSSION

In this study, on 32 boys, we found that the use of portable air filtration systems was associated with significant improvement in the quality of air and cardiovascular physiology. The use of AFS led to a fall in blood pressure, inflammatory, and DNA‐damage biomarkers. To our knowledge, this is the first study evaluating the impact of air pollution and the use of air filtration systems on the cardiovascular health of pediatric age‐group subjects.

The positive impact of short‐term use of portable air filtration systems has been shown in various previous studies (Fazlzadeh et al., 2022; Liu et al., 2021; Morishita et al., 2018; Shah et al., 2023). In summary, these studies have demonstrated positive effects on blood pressure and heart rate variability as well as on genetic markers, including DNA methylation, mRNA expression, and DNA metabolites. In a study exploring how particulate matter affects inflammatory pathways, Chen et al. found a positive correlation between particulate matter levels and the mRNA expression of inflammatory cytokines. In their study on 55 adults who spent over 75% of the time indoors with a baseline PM2.5 level of 46.8 μg/m^3^, the use of air purifiers led to a significant reduction in PM2.5 levels (8.6 μg/m^3^). Air purifiers were used for 9 days followed by a washout period. Compared to baseline values, the mRNA expression of *IL1*, *TNF*, *TLR2*, and endothelin 1 positively correlated with higher PM2.5 levels. Conversely, the corresponding miRNAs that act as expression suppressors of these genes were significantly downregulated, suggesting their involvement in mediating the physiological effects of particulate matter air pollution (Chen et al., 2018). Though the authors were able to demonstrate that short‐term use of air purifiers was able to mitigate the adverse effects of air pollution, the stringent instruction to remain indoors (in the same dormitories) for over three quarters of the days remains impractical. Their previous research on 35 Shanghai adults, with 48 h of exposure to air purifiers, was able to demonstrate that PM2.5 levels correlated negatively with DNA methylation in genes associated with inflammation, coagulation, and vasoconstriction. Global methylation was also found to be significantly reduced with an increase in PM2.5 levels. It was hypothesized that hypomethylation and consequent increases in the expression of pro‐inflammatory and pro‐coagulation genes can be one of the mechanisms of the cardiovascular impact of particulate matter air pollution (Chen et al., 2016). Another study from Korea evaluated the impact of particulate matter on oxidative DNA damage among patients with coronary artery disease. Thirty‐eight subjects with a mean age of 65.4 years were provided with 2 weeks of true and sham air filtration sequentially, separated by 2 weeks of washout period. The baseline average outdoor PM2.5 level was 25 μg/m^3^. After adjusting for multiple variables, it was found that urinary 8‐oxoDG levels were significantly reduced in the active filtration phase as compared to sham filtration, and the magnitude of reduction correlated with the fall in PM2.5 levels (Eom et al., 2022).

Though the ideal biomarker for DNA damage is not known, using an HPLC assay for 8‐oxo DG and comparing the levels between smokers and nonsmokers, it has been demonstrated that it can be used as a reliable biomarker for oxidative DNA damage (Lettieri Barbato et al., 2010). Furthermore, a systematic review reported that comet assays, micronucleus tests, and γ‐H2AX assays consistently provide robust results in detecting genomic instability and DNA damage, particularly related to exposure to particulate matter air pollution (Kazensky et al., 2024).

The impact of air filtration systems on pediatric health remains underexplored. In a retrospective case control study on 1522 cases of childhood pneumonia, the use of air filtration systems was associated with a lower incidence of pneumonia (odd ratio 0.66; 95% CI: 0.50–0.86) (Zhao et al., 2024). Another study found that the frequency of rescue medication was significantly reduced while using air filtration; however, the asthma symptom score did not show any statistically significant improvement (Lee et al., 2020). Current guidelines also recommend that treating physicians may advise the use of environmental controls, including air filtration systems, to help mitigate the harmful effects of air pollution on these conditions (Seidman et al., 2015).

One of the considerable strengths of our study was the pragmatic design. Boys in our study were exposed to air filtration only during night‐time and almost the entire day; they were outdoors exposed to the poor quality air. We anticipate that, even in real‐world homes, the children are unguarded to particulate matter air pollution during school and playtime hours.

Despite being the pragmatic design, our study had some limitations. Firstly, being single‐centric research limits the generalizability to areas where air quality is otherwise better. Second, we did not keep a washout period between two interventions; however, as it was not a randomized controlled study and we did not aim to cross over, the washout period might not have made any impact on the final findings. Third, our study population was restricted to boys only. We were not able to study the impact of air pollution and subsequent use of air purifiers on girls. Fourth, even with the use of air filtration systems, we were not able to reach the WHO recommended limits of PM2.5. It reflects on the sheer volume of the particulate matter air pollution, especially during the peak season in certain parts of our country. Due to this, though PM2.5 levels were significantly reduced, they remained notably higher than the baseline values of air pollution from similar studies in other countries. To mitigate this, we could have used more or more powerful air filtration systems in each room.

In conclusion, in this prospective study on boys residing in an orphanage located in the vicinity of hazardous category particulate matter air pollution, we were able to demonstrate that portable air filtration systems can reduce air pollution significantly and improve cardiovascular and DNA damage parameters. The findings of our study highlight the need for larger, long‐term studies to accurately assess the impact of portable air filtration systems on pediatric cardiovascular health.

## AUTHOR CONTRIBUTIONS

GA and PKS were involved in conceptualization. PKS, AK, AA, and DC were involved in methodology. PKS, GA, and RB were involved in formal analysis. RR, AK, VR, SC, VS, and RB were involved in investigation. JGM, PKS, and RR were involved in writing. DC, JGM, and GA were involved in writing review. PKS and GA were involved in funding acquisition. DC, PKS, and RR were involved in resources. DC was involved in supervision.

## FUNDING INFORMATION

This research received intramural funding from the institution vide letter number RC/UHSR/2023/178‐92.

## CONFLICT OF INTEREST STATEMENT

None to declare.

## ETHICS STATEMENT

The protocol was approved by the BREC (Biomedical Research and Ethics Committee) of the institute vide letter no. UHSR/RPSAC/2023/148‐149 dated 13‐10‐2023.

## Data Availability

The anonymized individual patient data can be shared upon request to the corresponding author and permission of the institutional ethics committee.
